# Long-Term Clinical Effectiveness of Ustekinumab in Patients With Crohn’s Disease: A Retrospective Cohort Study

**DOI:** 10.1093/crocol/otaa061

**Published:** 2020-07-28

**Authors:** Takahiro Ito, Atsuo Maemoto, Takehiko Katsurada, Hiroki Tanaka, Satoshi Motoya, Nobuhiro Ueno, Mikihiro Fujiya, Toshifumi Ashida, Daisuke Hirayama, Hiroshi Nakase

**Affiliations:** 1 IBD Center, Sapporo Higashi Tokushukai Hospital, Sapporo, Japan; 2 Department of Gastroenterology and Hepatology, Hokkaido University Hospital, Sapporo, Japan; 3 IBD Center, Sapporo-Kosei General Hospital, Sapporo, Japan; 4 Division of Gastroenterology and Hematology/Oncology, Department of Medicine, Asahikawa Medical University, Asahikawa, Japan; 5 Department of Gastroenterology and Hepatology, Sapporo Medical University School of Medicine, Sapporo, Japan

**Keywords:** clinical effectiveness, ustekinumab, Crohn’s disease, positive predictors

## Abstract

**Background:**

This study clarifies the long-term effectiveness of ustekinumab based on real-life data from Japanese Crohn’s disease (CD) patients.

**Methods:**

A total of 137 patients were included, and 124 patients (90.5%) were exposed to anti-tumor necrosis factor-α agents.

**Results:**

The clinical remission rate at week 52 was 32.4% in moderate to severely active CD patients. The achievement of clinical remission for 8 weeks after ustekinumab therapy induction was associated with clinical remission at week 52. Ustekinumab persistence rate at week 104 was 81.4%.

**Conclusion:**

Ustekinumab is effective and persistent in CD patients with the previous treatment history of several biologics.

## INTRODUCTION

The prevalence of Crohn’s disease (CD) has increased over the past 20 years,^1^ and there are more than approximately 70,000 patients in Japan. Treatment of CD has changed dramatically with the emergence of anti-tumor necrosis factor (TNF) agents.^2^ Despite their effectiveness, approximately one third of patients do not respond to these agents at the start of treatment (primary nonresponse), and a secondary loss of response (LOR) is observed in 10%–50% of patients per year.^2, 3^ It has been acknowledged that intolerance to anti-TNF agents and the associated side effects are common problems in clinical practice. Therefore, other biologics with mechanisms different from those of anti-TNF agents have been developed.

Ustekinumab (UST; Janssen Biotech, Horsham, PA) is a new class of biologically developed monoclonal antibody specifically targeting the p40 subunit of IL-12 and IL-23; both IL-12 and IL-23 are proteins that are important in the regulation of the immune system, are understood to play a role in immune-mediated inflammatory disease, and have a different mechanism of action from anti-TNFα agents. Ustekinumab has been approved in Japan for use in moderately and severely active CD since 2017.

A Study to Evaluate the Safety and Efficacy of Ustekinumab Induction Therapy in Patients With Moderately to Severely Active Crohn’s Disease (UNITI-2), a phase 3 clinical trial, compared UST to a placebo and found a significant difference in the ability of UST to induce remission, improve patient quality of life, and decrease the levels of inflammatory markers compared to the placebo.^4^ Additionally, the Study to Evaluate the Safety and Efficacy of Ustekinumab Maintenance Therapy in Patients with Moderately to Severely Active Crohn’s Disease (IM-UNITI), a phase 3 study, indicated that only 2.6% of patients developed antibodies over the course of 52 weeks, leading to a much lower immunogenicity profile compared to anti-TNF drugs.^5^ Based on the study of safety in the Psoriasis Longitudinal Assessment and Registry trial in which UST was used to treat psoriasis, UST is also well tolerated, and there was no increased risk of malignancy or serious infections.^6^

The recent clinical and open-label trials regarding UST have produced encouraging data on the treatment of refractory CD. However, despite the promising short- and intermediate-term effectiveness, persistence, and safety of UST in patients with refractory CD, the long-term, real-world outcome of UST treatment has not been well studied.^7–14^ Furthermore, there has been no report of long-term results on the effectiveness and safety of UST in Asia. Here, we present a retrospective cohort of a large number of Japanese CD patients treated with UST in *P*rincipal research in the *H*okkaido *O*rganization *E*mphasizing *N*utritional and Therapeutic *I*mprovement to IBD Patients’ E*x*pectation (Phoenix) Study. Our aim is to explore the effectiveness and safety of UST in Japanese CD patients based on real-world evidence.

## METHODS

### Study Design and Population

This was an observational, retrospective multicenter study in Hokkaido, Japan. A total of 137 CD patients who started UST at 6 hospitals from June 2017 to September 2019 were included. The research protocol for this analysis was determined by the Phoenix Study Group and approved by the ethics committee of the Sapporo Medical University School of Medicine. This study was preregistered at the University Hospital Medical Information Network Center (UMIN 000035384, available at http://www.umin.ac.jp/ctr/).

### Data Collection

All patients were diagnosed with CD based on the criteria determined by the Japanese Ministry of Health, Labor and Welfare, and their ages ranged from 15 to 77 years. There were no exclusion criteria. The baseline characteristics collected from the medical records were age, sex, disease duration, disease location and disease behavior according to the Montreal Classification system, anal disease, history of CD surgery, previous and concomitant CD medication (including 5-aminosalicylic acid, enteral nutrition, corticosteroids, budesonide, immunomodulators, and anti-TNF therapy), Crohn’s Disease Activity Index (CDAI) score,^15^ C-reactive protein (CRP, mg/dL) level, serum albumin level (g/dL), and the Simple Endoscopic Score for Crohn’s disease (SES-CD).^16^ The symptomatic response to treatment was evaluated using the CDAI, and the objective response was evaluated using CRP and albumin levels. Follow-up data were collected at weeks 8, 16, 24, 52, and 104. The initial intravenous (IV) UST dose was weight-adjusted (260, 390, or 520 mg), and the first subcutaneous (SC) dose was 90 mg, given 8 weeks after the IV UST. Further SC UST was given subcutaneously at either an 8- or 12-week interval at the discretion of the on-site physicians. All patients with at least 8 weeks of follow-up from their IV UST were included in this study. Patients with active disease at baseline (eg, CDAI ≥220) were used to determine the effectiveness, while all patients were used to determine persistence and safety outcomes.

### Outcomes and Definitions

Baseline patient characteristics were collected. Outcomes were evaluated at weeks 8, 16, 24, 52, and 104 (after UST induction). The primary endpoint was the proportion of patients achieving clinical remission (CDAI ≤150) at weeks 8 and 52. The secondary endpoints were (1) the identification of factors predicting the achievement of clinical remission at week 52; (2) changes in CDAI scores, biomarker levels, and endoscopic findings; (3) UST persistence at weeks 52 and 104; and (4) the incidence of adverse events (AEs). Clinical remission was compared between anti-TNF-naive and anti-TNF-exposed patients and between patients with and without concomitant immunomodulatory treatments (methotrexate or thiopurines). Additionally, the predictors of clinical remission at week 52 were investigated. The follow-up duration was defined as the interval from the date of the initial IV UST to the last visit. Primary response was defined as a 70 point-response (defined as a reduction in the CDAI score ≥70 from baseline) or achievement of remission by week 24. Secondary LOR was defined as CDAI more than 150 during the course after achieving remission. Patients who discontinued UST treatment due to primary or secondary nonresponse, AEs, or patient request without long-term sustained remission were considered to have experienced treatment failure and classified as nonresponders. The safety outcome was the number of drug-related AEs. Additionally, patients were not included in the evaluation hospital visits if the information needed for this study was missing for a certain time point.

### Statistical Methods

Continuous variables are presented as the medians with interquartile ranges (IQRs). The Wilcoxon test was used for nonparametric, paired, continuous variables, such as changes in CDAI scores. The Mann–Whitney *U* test was used to compare variables. Categorical variables are presented as percentages and were compared by using Fisher’s exact test. Receiver operating characteristic (ROC) curve analysis was used to identify optimal cutoff values of predictors (eg, disease duration, CDAI score, CRP, and albumin) with maximum sensitivity and specificity for achieving clinical remission at week 52. Predictive variables were assessed by univariate and multivariate analyses using logistic regression. The results are expressed as odds ratios (ORs) and 95% confidence intervals (CIs). Multivariate analysis was performed on variables with a *P* value of less than 0.1 in univariate analysis using backward stepwise logistic regression. Significant differences between outcomes were indicated by *P* values less than 0.05. The Kaplan–Meier method was used to assess UST persistence. All statistical analyses were performed with EZR (Saitama Medical Center, Jichi Medical University, Saitama, Japan), which is a graphical user interface for R (The R Foundation for Statistical Computing, Vienna, Austria).

## RESULTS

### Baseline Characteristics

A total of 137 patients who were followed for at least 8 weeks were included in this retrospective cohort study. The baseline characteristics of the included patients are given in Table 1. The median age of the patients was 37 years. Eighty-six patients (62.8%) were male. The median disease duration was 12 years (range 0–36). The patients were followed for a median of 66.9 weeks (IQR: 44.1–101.4). Two thirds of the patients (n = 92/137, 67.2%) had ileocolonic disease, and 33.6% of the patients (n = 46/137) had a penetrating disease phenotype at the maximum extent of their disease course. About 42.3% of the patients (n = 58/137) had perianal disease. Half of the patients (n = 72/137, 52.6%) had undergone prior intestinal resections. One hundred twenty-four patients (90.5%) had exposed more than one previous anti-TNF agent. Moreover, 52 patients (38.0%) were exposed to 2 anti-TNFα antibodies, while 13 patients were biologically naive. The median CDAI score was 151 (IQR: 78.8–239.8), the median albumin level was 3.6 g/dL (IQR: 3.1–4.1), and the median CRP level was 0.34 mg/dL (IQR: 0.10–1.36). Seventy-eight patients (56.9%) had undergone endoscopy at the beginning of UST therapy, and the median SES-CD score was 11 (IQR: 7–18). Ustekinumab was given concomitantly with 5-aminosalicylic acid in 89 patients (65.0%), enteral nutrition in 40 patients (29.2%), corticosteroids or budesonide in 53 patients (38.7%), and immunomodulators in 68 patients (49.6%).

**TABLE 1.** Baseline Characteristics

Baseline Characteristics		N = 137
Age	Median (min–max)	37 (15–77)
Sex—male	N (%)	86 (62.8)
Disease duration, years	Median (min–max)	12 (0–36)
Follow-up duration, weeks	Median (min–max)	66.9 (8.0–124.7)
Disease location		
Ileum	N (%)	33 (24.1)
Ileocolonic	N (%)	92 (67.2)
Colon	N (%)	12 (8.8)
Disease behavior		
Inflammatory disease	N (%)	31 (22.6)
Stricturing disease	N (%)	60 (43.8)
Penetrating disease	N (%)	46 (33.6)
Perianal disease	N (%)	58 (42.3)
Prior intestinal resections		
None	N (%)	65 (47.4)
One	N (%)	31 (22.6)
Two or more	N (%)	41 (30.0)
Prior biologics		
1 or 2 anti-TNF agents	N (%)	124 (90.5)
2 anti-TNF agents	N (%)	52 (38.0)
Vedolizumab	N (%)	0 (0)
Disease activity		
CDAI score	Median (IQR)	151 (78.8–239.8)
Albumin, g/dL	Median (IQR)	3.6 (3.1–4.1)
CRP, mg/dL	Median (IQR)	0.34 (0.10–1.36)
Endoscopic activity		
SES-CD score	Median (IQR)	11 (7–18)
Concomitant medication		
5-aminosalicylic acid	N (%)	89 (65.0)
Enteral nutrition (≥900 kcal/day)	N (%)	40 (29.2)
Corticosteroids or budesonide	N (%)	53 (38.7)
Immunosuppressants	N (%)	68 (49.6)

**FIGURE 1.**
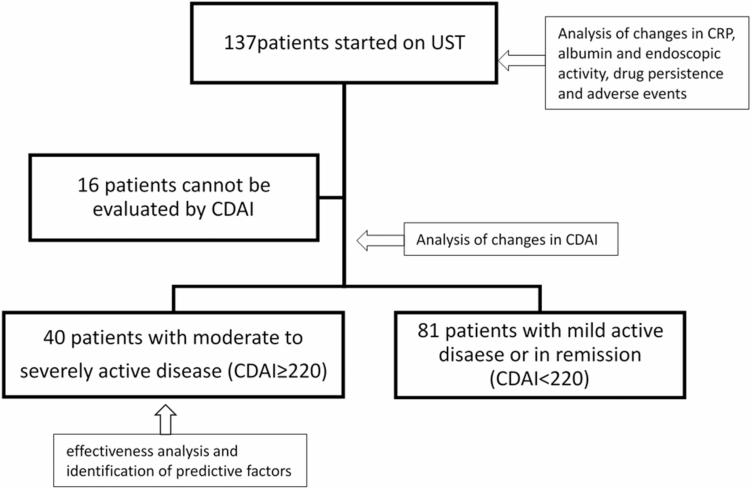
Details of the overall population at baseline.

Forty patients (n = 40/121, 33.1%) had clinically moderate to severely active disease (CDAI ≥220) at baseline (Fig. 1). Of the 81 patients with clinically mild or inactive disease (CDAI <220) at baseline, 49.4% (n = 40/81) had shown worsening of clinical symptoms and/or inflammatory biomarkers during medical treatments before UST, 25.9% (n = 21/81) had shown worsening endoscopic findings, and 21.0% (n = 17/81) had experienced side effects of previous biologic treatments. These patients were not included in the analysis of effectiveness. Of the patients with mild or inactive disease, 76.6% (n = 49/64, 17 patients did not reach week 52) maintained remission (CDAI ≤150) at week 52.

### Primary Endpoint: the Ratio of Clinical Remission Induced by UST

**FIGURE 2.**
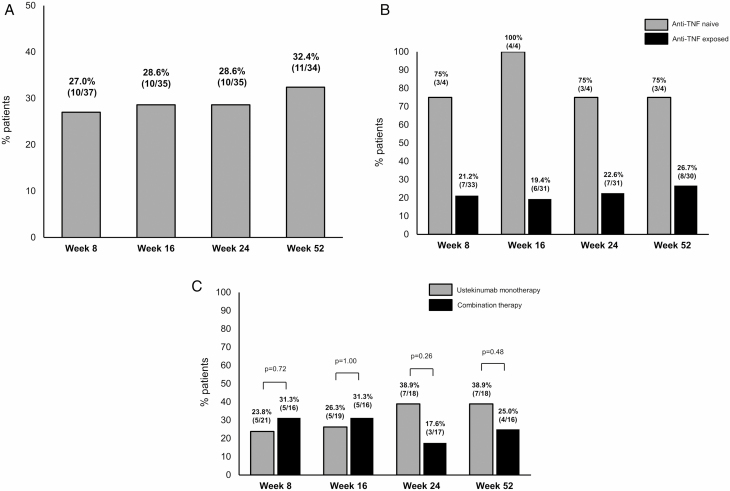
Clinical remission at weeks 8, 16, 24, and 52 in 63 patients with active disease. (A) Percentages of patients achieving clinical remission out of 40 clinically moderate to severely active CD patients treated with UST at weeks 8, 16, 24, and 52. (B) The clinical remission rate at week 52 was 75% in anti-TNF-naive patients and 26.7% in anti-TNF-exposed patients. (C) The clinical remission rate at week 52 was 38.9% in patients receiving UST monotherapy and 25.0% in those receiving combination therapy with immunomodulators.

At baseline, 40 patients had moderate to severely active disease (Fig. 1). The median CDAI score of these patients was 280.5. The proportions of patients achieving clinical remission at weeks 8 and 52 were 27.0% (n = 10/37) and 32.4% (n = 11/34), respectively (Fig. 2A).

### Secondary Endpoints

#### Identification of factors predicting the achievement of clinical remission at week 52

##### *Anti-TNF naive versus anti-TNF exposed*. 

Before starting UST, 36 patients (n = 36/40, 90.0%) with moderate to severely active disease at baseline had been exposed to anti-TNF, and 4 of them were anti-TNF naive. The proportions of patients achieving clinical remission at week 52 in anti-TNF-naive and anti-TNF-exposed patients were 75% (n = 3/4) and 26.7% (n = 8/30), respectively (Fig. 2B).

##### *Concomitant use of immunomodulators in patients with active disease.* 

At baseline, 17 patients (n = 17/40, 42.5%) initiated UST treatment while undergoing concomitant treatment with immunomodulators (thiopurines or methotrexate), while 23 (57.5%) initiated UST as monotherapy. The concomitant use of immunomodulators did not significantly contribute to the achievement of clinical remission at week 52 (monotherapy 38.9% [n = 7/18] versus combination therapy 25.0% [n = 4/16], *P* = 0.48; Fig. 2C).

The clinical factors associated with remission at week 52 are given in Table 2. Cutoff values of disease duration, CDAI score, CRP, and albumin were derived from ROC curve analysis (eg, cutoff value of disease duration was 9 with 78.3% specificity and 72.7% sensitivity, and area under the curve was 0.68 with 95% CI: 0.46–0.90). The OR of 2.42 of ileum for disease location means the OR which was compared to non-ileum. Also, the OR of 5.23 of inflammatory disease for disease behavior means the OR which was compared to noninflammatory disease. In univariate analysis, patients with a 70-point response at week 8 (OR: 10.4 [95% CI: 1.51–127.4]), clinical remission at week 8 (OR: 21.0 [95% CI: 2.57–202.4]), and an albumin level at least 3.8 mg/dL at week 8 (OR: 13.0 [95% CI: 1.83–163.7]) were more likely to achieve clinical remission at week 52. A long disease duration (>9 years) was a negative predictor of clinical remission at week 52 (OR: 0.11 [95% CI: 0.01–0.69]). Disease location and behavior were not associated with clinical remission. Prior intestinal resections and prior use of 2 anti-TNF agents tended to be associated with a decreased response but did not reach statistical significance as a univariate predictor.

**TABLE 2.** Univariate and Multivariate Predictors for Clinical Remission at Week 52

	Univariate Analyses			Multivariate Analyses		
	OR	95% CI	*P*	OR	95% CI	*P*
Age >37 years at inclusion	1.09	0.21–6.00	1			
Sex—male	0.65	0.12–3.61	0.709			
Disease duration, >9 years	0.11	0.01–0.69	0.008			
Disease location						
Ileum	2.42	0.27–22.3	0.363			
Ileocolonic	0.54	0.09–3.03	0.459			
Colon	1.05	0.08–9.08	1			
Disease behavior						
Inflammatory disease	5.23	0.76–44.4	0.079			
Stricturing disease	0.42	0.06–2.36	0.295			
Penetrating disease	0.59	0.08–3.40	0.705			
Perianal disease	1.58	0.30–9.54	0.715			
Anal fistula	0.59	0.08–3.40	0.705			
Prior intestinal resections	0.21	0.03–1.19	0.066			
Prior biologics						
Naive to anti-TNF agents	7.67	0.53–450.7	0.089			
2 anti-TNF agents	0.18	0.02–1.15	0.064			
Disease activity						
CDAI score >340 at baseline	0.52	0.04–3.59	0.682			
CRP >0.5mg/dL at baseline	2.82	0.50–20.8	0.271			
Albumin ≥3.8mg/dL at baseline	2.63	0.38–18.7	0.388			
SES-CD score >10 at baseline	0.53	0.06–4.21	0.659			
70-point response at week 8	10.4	1.51–127.4	0.007			
Clinical remission at week 8	21.0	2.57–202.4	<0.001	24.5	3.37–178.0	0.002
CRP >0.5mg/dL at week 8	0.29	0.04–1.67	0.141			
Albumin ≥3.8mg/dL at week 8	13.0	1.83–163.7	0.005			
Concomitant medication						
5-aminosalicylic acid	0.94	0.17–5.74	1			
Enteral nutrition (≥900 kcal/day)	0.89	0.15–4.84	1			
Corticosteroids or budesonide	1.83	0.35–10.2	0.475			
Immunosuppressants	0.53	0.09–2.82	0.477			

In the multivariable analysis, the achievement of clinical remission at week 8 remained a positive predictor of clinical remission 52 weeks after the induction of UST therapy.

#### Changes in CDAI, biomarkers, and endoscopic findings

**FIGURE 3.**
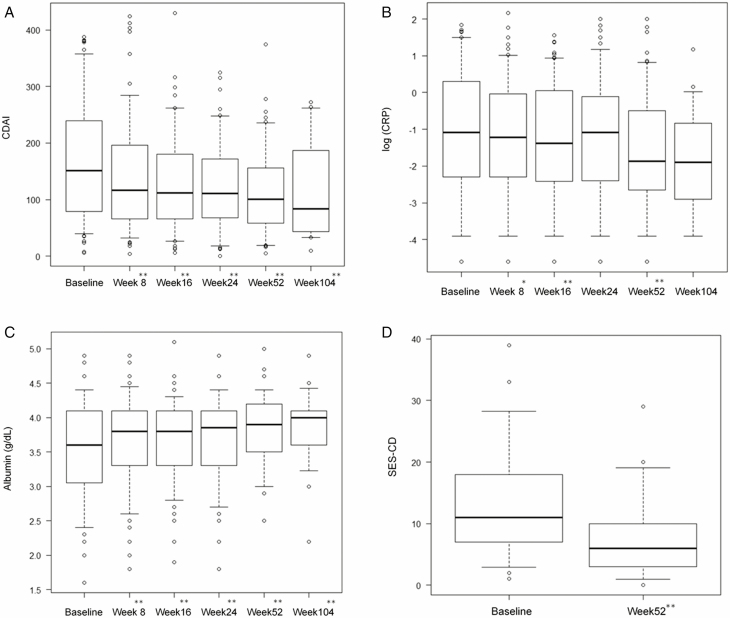
Outcomes for (A) CDAI scores, (B) CRP levels, (C) albumin levels, and (D) SES-CD scores over time in the total cohort. The outcomes for CDAI scores, CRP levels, albumin levels, and SES-CD scores over time in the cohort of treated patients. Box and whisker plots showing 5%–95% and median changes in (A) CDAI scores, (B) CRP levels, (C) albumin levels, and (D) SES-CD scores at baseline, week 8, week 16, week 24, week 52, and week 104. * and ** indicate statistically significant differences compared to baseline; *P* < 0.05 and *P* < 0.01, respectively.

CDAI scores and CRP level progressively decreased, and albumin levels significantly increased after UST treatment (Fig. 3A–C). We also examined changes in SES-CD scores in patients who underwent endoscopy at both baseline and week 52 (Fig. 3D, n = 40). The median SES-CD score was significantly reduced from 11 (IQR 7–18) at baseline to 6 (IQR 3–10) at week 52 (*P* = 0.049).

#### UST persistence at weeks 52 and 104

**FIGURE 4.**
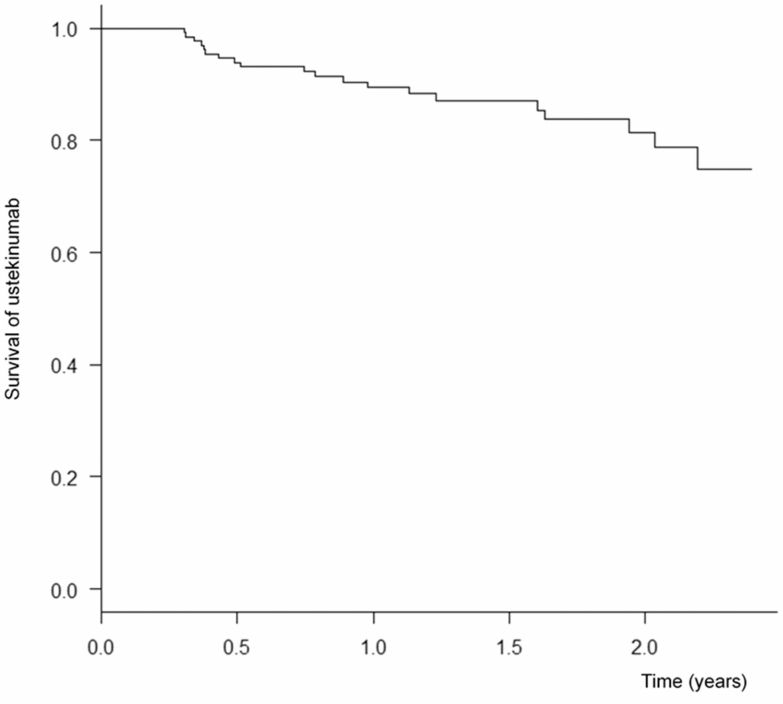
Kaplan–Meier survival curve for UST showing drug persistence. The cumulative probabilities for maintaining UST treatment at week 52 and week 104 were 89.4% and 81.4%, respectively.

Patients with primary nonresponse were 41.1% in moderate to severely active CD patients. On the other hand, patients with secondary LOR were 8.3% in moderate to severe patients in 1 year. As a result, 20 patients discontinued UST during the follow-up period in all populations (Fig. 4). All 20 patients discontinued due to primary nonresponse or secondary LOR and no patients stopped due to AEs. The cumulative probabilities for maintaining UST treatment at weeks 52 and 104 were 89.4% and 81.4%, respectively.

#### Incidence of AEs

In the entire study group, 13 patients experienced 18 AEs (Table 3). Abdominal pain and general fatigue were the most commonly described AEs. No deaths were recorded. All AEs were mild and did not require UST withdrawal. One 49-year-old male discontinued UST due to primary nonresponse. Then, ileal resection was performed, and ileal cancer was found in the resected specimen. We think this carcinogenesis was not an AE related to UST therapy.

**TABLE 3.** Adverse Events

Adverse Event (13 patients)	Events, n
Any adverse event	18
Abdominal pain	4
General fatigue	4
Worsening of arthralgia	2
Any adverse event at the subcutaneous injection site	5
Redness	2
Pain	2
Itching	1
Rash	1
Nausea	1
Ileal cancer	1

## DISCUSSION

This is the first real-world, multicenter study to evaluate the short- and long-term data on the effectiveness of UST in a large cohort of Japanese CD patients. The present study demonstrated that the clinical remission ratios in moderate to severely active CD patients at weeks 8 and 52 were 27.0% and 32.4%, respectively. We found that the achievement of clinical remission at week 8 was associated with the achievement of clinical remission at week 52. Additionally, UST persistence at weeks 52 and 104 were 89.4% and 81.4%, respectively. Taken together, in accordance with the UNITI study, our real-world data indicate the use of UST as an effective and safe treatment for the induction and maintenance of remission in active CD patients despite their history of exposure to biological therapies.

The clinical response is the most important outcome for evaluating the effectiveness of newly developed drugs. A phase 3 study of UST, UNITI-1, showed that the clinical remission rates at weeks 8 and 52 were 20.9% and 41.1%, respectively.^4^ Real-world observational studies showed that clinical remission rates at week 8 varied from 8% to 47% and those at week 52 ranged from 14% to 39.4% because open, unblinded studies are prone to bias.^7–9, 11, 12^ In our cohort, the clinical remission rate at week 8 (27.0%) and week 52 (32.4%) was almost equivalent to those reported in previous studies, including the UNITI trials. In the analyses of other endpoints, we identified the factors predicting the achievement of clinical remission at week 52. In univariate analysis, patients with a 70-point response in the CDAI score at week 8, clinical remission at week 8, and an albumin level at least 3.8 mg/dL at week 8 were identified as favorable predictors, while a long disease duration was a negative predictor of clinical remission at week 52. In multivariate analysis, the achievement of clinical remission at week 8 was identified as a favorable predictor of clinical remission at week 52. One cohort study, similar to the results of our study, found that the induction of remission at week 8 was a positive predictor of the maintenance of remission at 1 year.^11^ Because the serum concentration of UST is thought to be sufficient at week 8, it appears to be reasonable to predict the long-term response based on the activity at week 8. In contrast to the above predictors, the present study was the first to identify a high level of albumin, which is a marker of nutritional status, at week 8 as a positive predictor of the success of UST treatment in univariate analysis. Regarding the nutritional status, 2 cohort studies found that a high body mass index (BMI) was associated with clinical remission at 1 year in multivariable analysis.^7, 12^ These data suggest that the markers of patient nutritional status at week 8, including serum albumin, can be predictors of the effectiveness of UST treatment in clinical practice because the serum albumin level and BMI are routinely measured in most hospitals. In contrast, a long disease duration is thought to be associated with irreversible intestinal damage, including strictures and fistula. These 2 factors are considered generally negative predictors of success, regardless of the biologic treatments used.

Additionally, the present study showed that UST was effective in patients regardless of their previous exposure to biologics for induction and maintenance therapy, similar to the results of 3 real-world studies.^8, 11, 12^ Our study and other previous real-world studies included few anti-TNF-naive patients; therefore, the statistical power to determine the difference in UST effectiveness between patients who were anti-TNF-naive and anti-TNF-intolerant is thought to be insufficient. Further real-world analyses are warranted to clarify the effectiveness of UST in anti-TNF-naive patients.

The significance of the concomitant use of immunomodulators in UST treatment is still controversial. The present study showed that there were no predictive factors associated with UST failure-free persistence at 12 months in univariate and multivariate analyses. One report suggested that the concomitant use of immunomodulators may contribute to maintaining long-term persistence,^13^ whereas another study did not find such a benefit. Ustekinumab is more humanized than chimera IFX or ADA. The UNITI trials did not support a superior clinical outcome for patients treated with combination therapy compared with those treated with monotherapy. However, we must recognize the limitations of clinical trial data because there have been no prospective studies adequately powered to clarify the superiority or equivalence of combination therapy to monotherapy for each biologic drug. Whether combination therapy is beneficial depends on factors related to patient background, including disease phenotypes and medical histories. Considering the possibility of synergistic effects from combination therapy beyond altered immunogenicity or pharmacokinetics, the initial combination of UST with immunomodulators for several months as an induction therapy could be promising. Therefore, further exploration is needed to determine the significance and safety of combination treatment with UST for maintenance therapy.

The disease activity of CD is evaluated objectively based on endoscopic findings, calprotectin levels, ultrasound, and MRI. Among them, endoscopic evaluation is very important because accumulating evidence has suggested that healed mucosa contributes to better long-term outcomes of CD. Therefore, we evaluated endoscopic activity with the SES-CD score at baseline and week 52 in this study. In the UNITI studies, the results of the endoscopic subanalysis showed that the mean SES-CD score was reduced by 2.8 points for 8 weeks after a single UST infusion.^17^ One real-world cohort study showed that the median SES-CD score was significantly reduced from 12 to 3 at week 16.^10^ However, there have been no studies regarding endoscopic activity after long-term UST administration. In the present study, we showed a significant decrease in the median SES-CD score from 11 (IQR 7–18) at baseline to 6 (IQR 3–10; *P* = 0.049) at week 52, suggesting that UST treatment is promising with regard to maintaining both endoscopic and clinical remission for a long period of time.

Regarding UST persistence, we demonstrated excellent UST persistence rates at weeks 52 and 104 of 89.4% and 81.4%, respectively. In other real-life cohorts, treatment persistence rates ranged from 58.5% to 81.8% in 1 year.^7, 9, 11–14^ Only the study by Wils et al^14^ showed a 2-year persistence rate of 66%. Our results showed a relatively high rate even at 2 years after UST treatment. We believe that the reason for the high UST persistence rate in our cohort may be due to the lower disease activity at baseline or relatively higher proportion of CD patients naive to biologics in this cohort compared with those in other cohorts.

In our study, the frequency of AEs was low (9.5%, n = 13/137), and severe AEs requiring UST withdrawal were very unusual. In other studies, the most common AEs were dermatological disorders, systemic infections, and local abscesses.^7–14^ Concomitant therapy with corticosteroids or immunomodulators can increase the risk of AEs, especially infections.^18^ The safety of UST was evaluated in a Japanese subgroup in the UNITI studies.^19^ No patients had serious infections associated with IV UST in the Japanese subgroup, whereas only 2 patients had serious infections associated with SC UST during maintenance. No infusion reactions and deaths were reported through week 44 in the Japanese subgroup in those studies. The long-term data from randomized controlled trials evaluating psoriasis patient tolerance of UST after up to 5 years of follow-up did not show any increased risk of death, severe infection, or malignancy.^6^ Based on data from our cohort and previous data, UST treatment is considered to be relatively safe.

This study has some limitations. First, because of the retrospective multicenter design, the clinical and biochemical assessments and endoscopic evaluations could have been influenced by the different clinicians performing the evaluations in each institute, although the same diagnostic criteria and therapeutic guidelines were used in all institutions. Second, UST was probably continued without clinical response in some patients previously exposed to several biologics because there were few other therapeutic options in the current situation. Also, this patient bias in this cohort contributed to failing to analyze how anti-TNF exposure affects clinical induction and sustained remission by UST treatment. Third, the data on UST trough levels and antidrug antibodies were not available. However, recent data showed that monitored UST drug concentrations were not associated with the clinical outcome.^7, 20^

In conclusion, this is the first study to show the real-world effectiveness and safety of UST in a large cohort of CD patients in Japan. This study confirms the high degree of effectiveness and persistence of UST in CD patients previously exposed to several anti-TNF agents. The achievement of clinical remission at week 8 could contribute to better clinical outcomes. Ustekinumab was well tolerated in this long-term study.

## Data Availability

The data of this study are available on request due to privacy requirements.

## Notes

Funding: This work was supported by Health and Labour Sciences Research grants for research on intractable diseases from the Ministry of Health, Labour and Welfare of Japan (Investigation and Research for intractable Inflammatory Bowel Disease to H.N.) and Japan Society for the Promotion of Science (JSPS) Grants-in-Aid for Scientific Research (KAKENHI) grant number JP18H02799 (to H.N.). In addition, this work was partially supported by Grants-in-Aid for Scientific Research, 26460956 (to M.F.), Translational Research Network Program of Japan Agency for Medical Research and Development, C40 (to M.F.), and Development and Intractable Disease Health and Labour Sciences Research grants from the Ministry of Health, Labour and Welfare.

Author Contributions: T.I., A.M., N.U., M.F., and H.N. developed the study design. All authors collected data, and T.I. analyzed the data. T.I., A.M., M.F., and H.N. drafted the manuscript. All authors revised the manuscript and approved the final version of this manuscript.

Conflict of Interest: T.I., A.M., T.K., N.U., T.A., and D.H. have no conflicts of interest to declare. H.T. has received lecture fees from JIMRO Co., Ltd, AbbVie GK, EA Pharma Co., Ltd, Mochida Pharmaceutical Co., Ltd, Kyorin Pharmaceutical Co., Ltd, Mitsubishi Tanabe Pharma Corporation, Takeda Pharmaceutical Co., Ltd, Zeria Pharmaceutical Co., Ltd, and Janssen Pharmaceutical K.K. S.M. has received lecture fees from Mitsubishi Tanabe Pharma Corporation, Takeda Pharmaceutical Co., Ltd, and grants from EA Pharma Co., Ltd, AbbVie GK, Takeda Pharmaceutical Co., Ltd, Pfizer Inc. M.F. reports grants and personal fees from Nippon Kayaku Co., Ltd, grants, personal fees, and nonfinancial support from EA Pharma Co., Ltd, personal fees from Pfizer Inc., grants and personal fees from Takeda Pharmaceutical Company Ltd, grants and personal fees from Daiichi Sankyo Company, Ltd, personal fees from OLYMPUS Co., Ltd, personal fees from Janssen Pharmaceutical K.K., personal fees from KYORIN Pharmaceutical Co., Ltd, grants from AstraZeneca, personal fees from Tomakomai Minpo Co., Ltd, personal fees from Meiji Seika Pharma Co., Ltd, grants and personal fees from Mochida Pharmaceutical Co., Ltd, personal fees from Mitsubishi Tanabe Pharma Corporation, grants and personal fees from Otsuka Pharmaceutical Co., Ltd, grants and personal fees from Kyowa Hakko Kirin Co., Ltd, personal fees from Zeria Pharmaceutical Co., Ltd, personal fees from Asahi Kasei Corporation, grants and personal fees from Eisai Co., Ltd, grants from GlaxoSmithKline K.K., grants from AbbVie Inc., grants from Sumitomo Dainippon Pharma Co., Ltd, grants from ASKA Pharmaceutical Co., Ltd, grants from Boston Scientific Corporation or its affiliates, grants and nonfinancial support from Kamui Pharma, Inc., outside the submitted work. In addition, M.F. has a patent Intestinal Protectant (JP 5660508) issued, and a patent Intestinal Protectant (EP 2559437) issued. H.N. reports receiving personal fees from Abbvie Inc., Kissei Pharmaceutical Co., Ltd, Kyorin Pharmaceutical Co., Ltd, Mitsubishi Tanabe Pharma Corporation, Janssen Pharmaceutical K.K., Takeda Pharmaceutical Co., Ltd, Pfizer Inc., Cell gene Corporation, EA Pharma Co., Ltd, Zeria Pharmaceutical Co., Ltd, Mochida Pharmaceutical Co., Ltd, Nippon Kayaku Co., Ltd, Daiichi Sankyo Company, Ltd, JIMRO Co., Ltd, Kissei Pharmaceutical Co., Ltd, as well as grants for commissioned/joint research from Hoya Group Pentax Medical, Boehringer Ingelheim GmbH, and Bristol-Myers Squibb Company.
